# Rinaldo Bellomo's seminal contribution to observational research using the ANZICS CORE registry

**DOI:** 10.1016/j.ccrj.2025.100140

**Published:** 2025-10-14

**Authors:** Michael Bailey, Sean M. Bagshaw, Graeme K. Hart, David Pilcher

**Affiliations:** a Australian and New Zealand Intensive Care Research Centre, School of Public Health and Preventive Medicine, Monash University, Melbourne, Victoria, Australia; b Department of Critical Care Medicine, Faculty of Medicine and Dentistry, University of Alberta and Alberta Health Services, Edmonton, Canada; c Department of Intensive Care Medicine, Austin Health, Heidelberg, Victoria, Australia; d Centre for Digital Transformation of Health, The University of Melbourne Faculty of Medicine, Dentistry and Health Sciences, Melbourne, Victoria, Australia; e Department of Intensive Care, Alfred Health, Melbourne, Victoria, Australia; f Australian and New Zealand Intensive Care Society (ANZICS) Centre for Outcome and Resource Evaluation, 101 High Str, Prahran, Victoria 3004, Australia

**Keywords:** Critical care, Registry, ANZICS CORE, Adult patient database, Observational research, Trial design

## Abstract

Rinaldo Bellomo advanced critical care not only through randomised trials but also through rigorous use of observational data, particularly from the ANZICS Centre for Outcome and Resource Evaluation (ANZICS CORE) Registry. At a time when retrospective analyses were often confined to hypothesis generation, he showed that carefully curated, clinically grounded registry studies could inform policy and change practice. Recognising early the potential of ANZICS CORE to become a leading registry, he worked to strengthen its data architecture and published in journals such as *The New England Journal of Medicine* and *JAMA*, helping to spark global dialogue and shape guidelines. Using the Adult Patient Database, he described epidemiological trends, identified clinically relevant questions, designed, justified and evaluated randomised trials, and monitored the uptake of evidence-based practice. His work addressed key challenges in sepsis, acute kidney injury, glycaemic control, temperature management and health equity, and was marked by clear case definitions, extensive sensitivity analyses and transparent reporting. This article reviews selected contributions using ANZICS CORE data and outlines how his legacy endures through the value of these datasets and the many researchers he mentored.

## 1 Introduction

Professor Rinaldo Bellomo is widely regarded as the foremost critical care researcher of his generation. With more than 2000 peer-reviewed publications and leadership of numerous randomised trials, his methodological range was exceptional. A hallmark of his career was sustained observational research using ANZICS CORE datasets, including the APD, Critical Care Resources and paediatric registries. This work became a powerful driver of policy and practice change and established registry-based infrastructure as a foundation for critical care research programs.

While many viewed large-scale registries as tools for audit or descriptive reporting, Rinaldo recognised their potential to generate clinically meaningful, policy-relevant evidence and to track the uptake of evidence-based recommendations. He championed the idea that carefully curated data, analysed with rigour and clinical insight, could drive discovery, inform guidelines, and shape international best practice.

The early development of ANZICS CORE focused on design, implementation, data quality, the proportion of ICUs contributing data, financial stability, and clinical quality assurance. From the early 2000s he worked with the CORE committee and staff to refine the data architecture so that disease- and treatment-specific elements could be added rapidly to the baseline dataset.

Rinaldo focused on the APD, one of the world's most comprehensive critical care datasets, which now contains more than 3.5 million adult ICU admissions in Australia and New Zealand.^1^ The APD has underpinned hundreds of research publications, audits, and quality improvement initiatives. For Rinaldo, it was not only a system-level resource but also a dynamic research platform that enabled scalable, context-specific inquiry. When clinical trials took years, the APD could yield insights within days. His foundational 2006 article formalised this vision and laid the groundwork for a sustained, practice-shaping programme of research.^2^

Collaboration was central to his approach. He worked closely with the ANZICS Clinical Trials Group, using registry insights to support study design, inform sample size, feasibility assessments, implementation, and evaluation.

Rinaldo's work spanned bench, animal, and clinical research, translating basic science into patient care. His interests included catecholamine responsiveness in sepsis and acute kidney injury; respiratory failure and support strategies; neurologic injury and recovery after global ischaemia and hypoxaemia; metabolic disturbances and their impact on survival; and the quality of life and resource utilisation of frail older adults. He challenged preconceptions and helped establish new, evidence-based treatment paradigms. The following sections highlight major areas where he reshaped understanding and practice.

## 2 Acute kidney injury and the RIFLE-AKIN paradigm

Much of Rinaldo's work focused on acute kidney injury (AKI), recognising it as a poorly understood and defined, underappreciated complication of critical illness. In close collaboration with Sean Bagshaw, a rising leader in nephrology and intensive care, he helped launch a practice-shaping series of APD-based studies examining AKI incidence, classification, and outcomes in ICU patients. In 2007, Sean, Rinaldo and others published a study in *Critical Care* showing that early AKI was common and strongly associated with adverse outcomes in Australian ICUs.^3^ This was followed in 2008 by a second *Critical Care* publication linking early AKI to sepsis, which became one of the most cited observational studies of AKI and sepsis globally.^4^ That same year, two complementary studies in *Nephrology Dialysis Transplantation* evaluated the RIFLE and AKIN classification systems, offering early large-scale validation of these frameworks across thousands of ICU admissions.^5^^,^^6^ Collectively, these four publications have been cited more than 2000 times.

This body of work demonstrated how standardised clinical definitions could be applied to large-scale registry data to produce clinically meaningful and foundational knowledge of AKI epidemiology, injury staging, and prognostic classification systems. These analyses also clarified the intersection of sepsis and AKI, revealing distinct patterns in septic versus non-septic renal failure.

## 3 Glycaemic control and glucose variability

Continuing his partnership with Sean Bagshaw, Rinaldo extended his focus to glycaemic control in the critically ill. In 2009, they published two influential studies using the APD, one in *Critical Care* and the other in *Critical Care Medicine*.^7^^,^^8^ Rinaldo was among the first to question the prevailing enthusiasm for tight glucose control following the Leuven trials. These analyses demonstrated that both early hypoglycaemia and high intra-patient glucose variability were independently associated with increased mortality.

The findings from these large-scale observational studies complemented the results of the NICE-SUGAR trial,^9^ in which Rinaldo was a key investigator. Together, the APD and trial evidence reinforced concerns that tight glycaemic control was not beneficial and might be harmful. This convergence of real-world and randomised data contributed to updated guidelines recommending more moderate glucose targets and stronger safeguards against hypoglycaemia, highlighting the APD's capacity to inform timely and policy-relevant questions and influence international practice.

## 4 H1N1

During the 2009 H1N1 influenza pandemic, recognising that Australia and New Zealand would experience the winter wave ahead of the Northern Hemisphere, Rinaldo together with Steve Webb, Andrew Davies, Simon Finfer and others collaborated to publish two landmark studies in *The New England Journal of Medicine* and *JAMA*, that characterised the ICU burden of H1N1 and the early use of extracorporeal membrane oxygenation.^10^^,^^11^ Data collected by clinicians at sites throughout Australia and New Zealand were combined with information from the APD to provide critical early insights that shaped international preparedness. This solidified ANZICS-led research as a credible and timely contributor to global discourse and exemplified Rinaldo's drive to align evidence generation with emerging clinical priorities.

## 5 Lactate and risk stratification in critical illness

In the early 2010s, Rinaldo collaborated with Alistair Nichol, Jamie Cooper and others to examine the prognostic value of lactate in ICU patients. They used information that combined data from electronic medical records and the APD to reframe how risk is understood in critical illness. Their 2010 study showed that even modest elevations in lactate, termed relative hyperlactatemia, were independently associated with increased hospital mortality.^12^ A follow-up study in 2011 demonstrated that dynamic lactate trends over time also carried strong prognostic value, independent of baseline levels.^13^

Together, these studies challenged the reliance on static lactate thresholds and established lactate kinetics as a meaningful signal in ICU risk assessment. As always, Rinaldo subjected these findings to exhaustive sensitivity analyses before endorsing them for publication. Their influence stemmed not only from the findings themselves, but from the analytical rigour that defined Rinaldo's approach to registry-based analysis. In turn, this led to the incorporation of lactate as a routine component of the APD and its inclusion in baseline ICU risk prediction models.

## 6 Trial design and statistical foundations

Rinaldo also shaped the statistical foundations of trials in intensive care. He argued that unit-level interventions are vulnerable to contamination and secular trends if individually randomised, and he advocated cluster randomised crossover designs in which each ICU serves as its own control.^14^ He then worked with methodologists to provide practical power and sample size methods for such trials in Australian and New Zealand ICUs, using parameters informed by registry data.^15^ This work helped turn the APD from a descriptive resource into a planning tool for efficient, registry practice-embedded trials.

## 7 Oxygen and carbon dioxide: from observational insights to interventional trials

Between 2011 and 2013, Rinaldo and colleagues published a series of influential observational studies examining the relationship between arterial blood gases and outcomes following cardiac arrest. The first in 2011, found that arterial hyperoxia was independently associated with increased in-hospital mortality among post-resuscitation patients.^16^ This challenged prevailing assumptions that supraphysiological oxygen levels were benign or even protective, prompting a global reassessment of oxygen therapy targets.

Building on this work, a 2012 study by Rinaldo and Glenn Eastwood expanded the analysis to a broader cohort of mechanically ventilated ICU patients.^17^ It revealed a U-shaped relationship between PaO_2_ and mortality, suggesting that both hypoxia and hyperoxia posed risk. In 2013, together with Antoine Schneider, he shifted focus to arterial carbon dioxide tension (PaCO_2_), showing that both hypocapnia and hypercapnia were associated with increased mortality in post-cardiac arrest patients.^18^

These high-quality, APD-linked studies provided the empirical rationale for the TAME (Targeted Therapeutic Mild Hypercapnia) trial, ultimately published in *The New England Journal of Medicine*.^19^ This progression from hypothesis generation using real-world registry data to global interventional clinical trial, epitomised Rinaldo's vision for how rigorous observational research could catalyse practice-changing evidence.

## 8 Temperature: associations, interventions and research translation

In 2012, Rinaldo collaborated closely with Paul Young, Manoj Saxena and others on an APD-based study that examined the association between early peak temperature and mortality, stratified by infection status.^20^ Moderate fever was associated with improved outcomes in infected patients but worse outcomes in non-infected patients. These findings directly informed the design of the HEAT trial, which tested paracetamol for fever control in critically ill adults with suspected infection.^21^

Five years later, following the publication of the Targeted Temperature Management (TTM) trial, Ryan Salter, Rinaldo and others reported an almost immediate increase in the average temperature of patients managed in Australian and New Zealand ICUs after cardiac arrest, as clinicians abandoned therapeutic hypothermia.^22^ Together, these examples illustrate how Rinaldo used the APD not only to generate timely, practice-relevant questions but also to measure how new evidence was implemented and influenced behaviour at the bedside.

## 9 Sepsis: defining, redefining and validating

Among Rinaldo's most influential contributions using the APD were a series of landmark studies that reshaped how sepsis was understood and classified. In 2014, he led a major study published in *JAMA* that analysed over 100,000 sepsis admissions across a decade, demonstrating a sustained decline in mortality from severe sepsis and septic shock in Australia and New Zealand.^23^ Cited more than 2000 times (more than any other APD article) the study reinforced the value of ANZICS CORE registry for evaluating outcomes at scale.

In 2015, together with Maija Kaukonen again, he co-led a pivotal *New England Journal of Medicine* study that critically evaluated the long-standing SIRS criteria.^24^ The analysis showed that SIRS lacked prognostic precision and frequently failed to identify patients with serious infection. This, together with the 2014 mortality findings, directly informed the development of the Third International Consensus Definitions for Sepsis and Septic Shock (Sepsis-3), published in *JAMA* in 2016.^25^

Recognising the need for timely real-world validation of the Sepsis-3 criteria, with Eamon Raith and others, Rinaldo led a multicentre APD-based study comparing the prognostic performance of SOFA, qSOFA, and SIRS among critically ill patients with suspected infection. Published in *JAMA* in 2017,^26^ it remains the most widely cited external validation of the Sepsis-3 criteria. Luregn Schlapbach, Rinaldo and others then went on to adapt this work to assess the validity of Sepsis-3 criteria in children using both adult and paediatric registry datasets.^27^

These achievements reflect more than scientific expertise, they reveal Rinaldo's deep strategic insight, alignment with emerging priorities, unmatched timing in delivering high-impact evidence when global frameworks were ready to evolve and an amazing ability to pull individuals together to rapidly create high quality research to immediately fill emerging gaps in scientific knowledge.

## 10 Persistent critical illness

Rinaldo also helped pioneer a new conceptual framework in intensive care: persistent critical illness (PerCI). Collaborating with Jack Iwashyna, he used APD data to define and characterise this state. Their 2016 study, published in *Lancet Respiratory Medicine*,^28^ showed that after approximately 10 days in ICU, the drivers of mortality shifted from acute physiological derangement to underlying chronic vulnerability.

This work reframed how Intensivists understand prolonged ICU trajectories, establishing PerCI as a distinct clinical phenotype with implications for care planning, resource allocation, and recovery pathways. As the first large, multicentre effort to define PerCI using population-level data, it has since become a cornerstone for subsequent research into long-stay ICU cohorts, rehabilitation strategies, and survivorship.

## 11 Frailty and age

Rinaldo advocated for frailty, rather than chronological age, as a more meaningful indicator of physiological reserve and prognosis. This perspective informed early APD-based work examining outcomes for very old ICU patients,^29^ which challenged assumptions about age-based triage and highlighted the need for more refined prognostic tools.

The subsequent integration of frailty into the APD was shaped in part by Rinaldo's long-standing collaboration with Sean Bagshaw, who drove adoption of the Clinical Frailty Scale in critical care worldwide.

In 2019, Rinaldo working with Jai Darvall, Ashwin Subramaniam and others, conducted a bi-national study using the Clinical Frailty Scale within the APD, demonstrating its prognostic value across older ICU cohorts.^30^ Frailty was independently associated with increased mortality, longer ICU stays, and a lower likelihood of discharge to home. These findings reinforced the clinical importance of frailty and reflected Rinaldo's enduring commitment to tailoring treatment through robust observational data.

## 12 Sex and gender

Among Rinaldo's most socially impactful research programs was Lucy Modra's doctoral work examining sex and gender disparities in intensive care. Drawing on over two decades of APD data, the project produced four landmark studies between 2021 and 2024.[31], [32], [33], [34] Collectively, the findings revealed that women were underrepresented in ICU admissions, received less organ support, and experienced different outcomes based on diagnostic patterns.

One study showed that mortality varied depending on whether the condition was more common in the opposite sex, suggesting that diagnostic familiarity may influence clinical decision-making. Another demonstrated that women were less likely to receive interventions such as mechanical ventilation or renal replacement therapy, even after adjusting for severity and diagnosis. The final article, published in *Chest*, was the first global registry-based study to explicitly identify and analyse ICU patients recorded as neither male nor female, revealing distinct clinical patterns and outcomes. This work also reflected Rinaldo's talent for mentoring emerging clinician-scientists with a diverse range of interests, many of whom are now research leaders in their own right.

## 13 Personal lessons from our friend and mentor Rinaldo

Responding to reviewers from top-tier journals such as *New England Journal of Medicine*, *JAMA*, and *The Lancet* requires clarity, transparency, and scientific precision. These journals demand not only originality and methodological rigour but also global clinical relevance. Reviewer critiques may challenge foundational assumptions, request complex re-analyses, or seek additional data that can be difficult to produce retrospectively. Rinaldo thrived in this environment. He frequently strengthened manuscripts during revision by linking study cohorts to the APD to extract robust outcome and risk-adjustment data. Many studies would not have been published in high impact journals without this approach.^35^^,^^36^

Rinaldo believed in simplifying the question before solving it. He was driven not just by clinical insight but by a deep instinct to teach, challenge, and clarify. He inspired belief and shared ownership in the work. His leadership did not stem from title or position, but from presence. He listened carefully, led with conviction, and never lost sight of the human meaning behind the numbers.

His approach to research echoed the philosophy of Karl Popper, who argued that scientific progress arises from efforts to disprove rather than confirm. Discovering a signal in a dataset marked the beginning of inquiry, not its conclusion. He worked methodically to test, refine, and, where necessary, dismantle the signal. Through subgroup analyses, multivariable modelling, and rigorous sensitivity checks, he interrogated each finding until it was both statistically credible and clinically robust. To Rinaldo, data were not merely evidence; they were hypotheses to be tested with integrity and rigor.

## 14 Rinaldo's legacy and the global benchmark

**Fig. 1:**
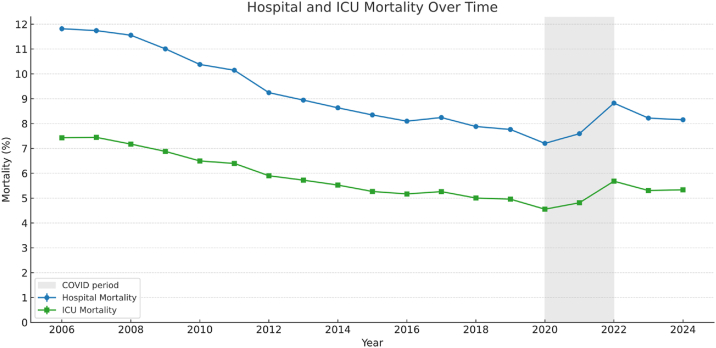
Adult Patient Database (APD) data from 2006 to 2024 showing annual hospital and ICU mortality in Australia and New Zealand.

Using ANZICS CORE data from the past two decades, Fig. 1 shows a sustained decline in ICU mortality across Australia and New Zealand. Current hospital mortality is around 8–10 %, placing these systems among the best internationally. This improvement not only reflects advances in care and scientific infrastructure but also the research culture that Rinaldo helped build. His mentorship, strategic vision and belief in the power of data left an enduring imprint on the field. He showed that, with the right questions and tools, observational data can move beyond description to transform practice. The research output from the APD and the global standing of the ANZICS CORE Registry remain lasting testaments to his vision. The discipline will face new challenges; his legacy provides both a foundation and a guide for the future. His rigour, enthusiasm, energy, clarity of thought, organisational skill and inclusive leadership remain legendary. He is greatly missed by colleagues, trainees and friends in Australia, New Zealand and worldwide. We offer our sincere condolences to his family.

## Ethics

Not applicable (narrative review using published/registry data).

## Credit authorship contribution statement

**Michael Bailey:** Conceptualisation, Methodology, Investigation, Data Curation, Writing Original draft, Writing Review and editing. **Graeme Hart:** Writing, Review and editing. **Sean Bagshaw:** Writing, Review and editing. **David Pilcher:** Conceptualisation, Writing, Review and Editing.

## Data availability

Not applicable.

## Funding

This research did not receive any specific grant from funding agencies in the public, commercial, or not-for-profit sectors.

## Conflict of interest

The authors declare no competing interests.
